# EfficientNet-driven deep learning for accurate detection of faults in photovoltaic cells

**DOI:** 10.1371/journal.pone.0342647

**Published:** 2026-04-03

**Authors:** Montaser Abdelsattar, Ahmed AbdelMoety, Mohamed A. Ismeil, Ahmed Emad-Eldeen

**Affiliations:** 1 Electrical Engineering Department, Faculty of Engineering, Qena University, Qena, Egypt; 2 Electrical Engineering Department, Faculty of Engineering, King Khalid University, Abha, Saudi Arabia; 3 Renewable Energy Science and Engineering Department, Faculty of Postgraduate Studies for Advanced Sciences (PSAS), Beni-Suef University, Beni-Suef, Egypt; Northeastern University, UNITED STATES OF AMERICA

## Abstract

The global transition to renewable energy resources has driven the need for potent international practice attention on optimized Photovoltaic (PV) system performance, particularly increased PV cell efficiency. Due to environmental conditions and manufacturing defects, the process of energy conversion through these cells may regress substantially. Early identification of these issues can be the key to improving maintenance procedures and extending the useful lives of solar panels. In this study, a robust Deep Learning (DL) framework based on the EfficientNetV2 architecture is presented to increase the accuracy of fault identification in PV cells. Three EfficientNetV2 variants—EfficientNetV2B0, EfficientNetV2B2, and EfficientNetV2M—were evaluated to identify the most effective model for this paper. This solution combines sophisticated techniques for image preprocessing and augmentation by employing a dataset of 2,500 images, including both defective and non-defective cells. This approach makes it possible for us to tailor the model, especially for the difficult process of anomaly detection. Among the assessed models, EfficientNetV2M exhibited the highest performance, achieving an overall accuracy of 89.6%, precision of 88.6%, recall of 77.5%, and F1-score of 82.7%, signifying superior generalization and learning capability. EfficientNetV2B2 demonstrated the highest validation accuracy of 82.4% in the feature extraction phase, indicating strong consistency throughout training. The ability to accurately detect both nuanced and overt issues underscores the suitability of the EfficientNetV2-based approach for the preventive maintenance of PV systems.

## 1. Introduction

### 1.1. Background information

Photovoltaic (PV) cells are essential elements of solar energy systems. They operate by directly transforming sunlight into electricity using the PV effect. Upon collision with these cells, photons excite electrons, generating an electric current devoid of mechanical components, noise, or harmful discharges [1]. Progress in PV technology—including advances in silicon, perovskite, and organic solar cells—is increasing efficiency and reducing manufacturing costs, hence enabling more economically viable solar power [2]. Solar energy is as much a part of the technology as it is to achieve sustainable development. Solar power, a clean and sustainable energy source, helps reduce dependence on fossil fuels and greatly reduces greenhouse gas emissions [3]. Secondly, it promotes economic development and decreases energy poverty, especially in the poorest parts of the world, by providing easier access to energy in remote areas [4]. This means that ensuring PV technology is integrated into the world’s energy infrastructure is essential for achieving a sustainable and secure future.

In real-world PV deployments—particularly utility-scale solar farms and large rooftop installations—undetected cell-level defects can lead to measurable energy yield losses, accelerated module degradation, and unplanned maintenance actions. Localized hotspots, microcracks, irregularities induced by the soil, and other degradation effects may not seem obvious at first glance, but they could develop over time, and lead to mismatches at the string level, reduced power production, and extended outages. Therefore, preventive maintenance is based on a precise and automated defect detection methodology that allows earlier intervention, the reduction of the burden of manual inspection, the control of operational disruptions, and finally, the cost effectiveness and reliability of photovoltaic assets.

**Fig 1:**
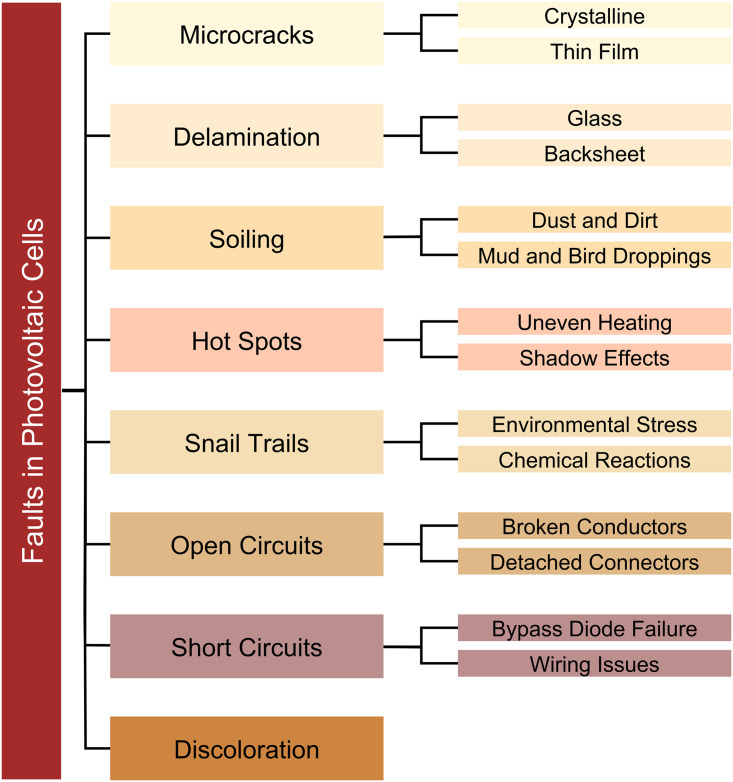
Classification of common faults in PV cells.

Microcracks pose a potential risk to both the structural stability and performance of solar cells, whether they’re crystalline or thin-film. These tiny fractures usually occur due to mechanical stress or manufacturing defects [5]. Delamination is the process in which the layers inside a PV module, such as the glass or backsheet, become detached from each other. This segregation not only diminishes the module’s strength, but also diminishes its longevity and efficiency [6]. PV cells undergo a substantial decline in performance when they get dirty as a result of the buildup of dust, dirt, or bird excrement on the panels’ surface [7]. Unequal distribution of heat or the presence of shadow effects may lead to the formation of localized areas of high temperature in solar cells. If not handled, these hot spots can deteriorate and reduce the overall efficiency of the solar panel [8]. The aesthetic and practical attributes of solar panels are degraded by visible streaks on the panel surface, mostly induced by chemical reactions or environmental stress [9]. Electrical issues such as short circuits and open circuits may lead to significant power losses if they are caused by broken cables, disconnected connections, or failures in bypass diodes [10]. Discoloration brought on by ageing or exposure to severe weather conditions reduces the panels’ capacity to effectively absorb sunlight, hence lowering their output [11]. A comprehensive visual representation presented in Fig 1 of these categories, which outlines the many types of faults and their respective subcategories in solar systems.

Considering the heterogeneity of PV faults and the influence of environmental factors, defect identification in PV systems is a challenging task. Defects include: small cracks, separation of layers, or zones of less than maximum sunlight exposure, all of which often produce minuscule changes in electrical or physical properties, which the normal differentials due to temperature and light intensity tend to mask. The joint complexity of different defects existing in a system and their very different manifestations makes detection difficult; it is tough to discriminate between normal fluctuations in power generation due to environmental conditions and real failures where a fault is present. Moreover, environmental variations can significantly influence the performance of fault detection designs. To do that, it is necessary to apply advanced data processing algorithms to filter out the real issues from phony alarms [12].

It is difficult to overstate the importance of rapid defect detection. Rapid defect identification and repair is crucial to ensure performance and maximize the lifetime of PV systems. Detecting problems in a timely manner is crucial in preventing minor faults from escalating into major breakdowns, thereby safeguarding capital invested in solar systems and ensuring continuous power generation. Predicting defects and taking action before they do a significant amount of damage may be a lot more effective at reducing maintenance costs and downtime, increasing the overall reliability and performance of solar power systems [13].

Traditional approach to fault detection in PV cells is more of a cumbersome process that involves human inspection, sometimes using specialized equipment. These are simple but labour intensive processes, limited to the subjective judgement of the inspector – a human being physically at the site. Infrared imaging: one of the important method for detecting abnormality such as hot spots or interior flaws which cannot be seen with the human eye [14].

Regarding automation, there are now systems in place that use less complex Machine Learning (ML) models or rule-based algorithms to simplify the detection process. These systems have the ability to automatically detect and classify problems, such as shading, soiling, or deterioration, by analyzing patterns in data that may not be detected during hand inspections. Nevertheless, these automated systems often encounter constraints such as elevated rates of incorrect positive identifications and challenges in handling intricate fault situations in diverse environmental settings. Certain systems include rudimentary ML approaches in order to enhance precision; however, they continue to have challenges in achieving full fault coverage [15].

DL methods are gaining increasing popularity because of their ability to effectively scan large datasets and identify subtle patterns indicative of problems. Convolutional Neural Networks (CNNs) have been used in automated PV diagnostics to accurately analyze Electroluminescence (EL) images of PV cells and detect anomalies such as microcracks [16]. Despite the improvements, these systems often need substantial quantities of data and processing capacity, and their effectiveness may be hindered by models that lack generalization across different PV installations or by inadequate training data [17].

The use of Internet of Things (IoT) technology has also enhanced fault detection systems by facilitating the gathering and analysis of data in real-time, a critical factor in promptly identifying faults and minimizing efficiency declines. IoT systems have the capability to constantly monitor a diverse range of metrics, enabling prompt reactions to signals of faults [18].

DL is a significant breakthrough in the field of identifying defects in PV systems using image-based techniques. DL is an effective choice for detecting and diagnosing different flaws because it uses sophisticated algorithms that can understand complex visual data from PV cells. These include a diverse array of issues, spanning from minor cracks and separation to more nuanced challenges such as shading effects and specific regions of elevated temperature. DL has a greater ability than typical defect detection approaches to extract features from complicated and extensive image datasets. This makes it crucial for ensuring the quality and reliability of PV systems [19,20].

EfficientNet is an ideal choice for this purpose because of its optimized design that achieves a balance between efficiency and accuracy, making it well-suited for DL. EfficientNet exhibits consistent scaling in terms of depth, breadth, and resolution, guaranteeing optimal performance over a wide range of image datasets without the need for unnecessary processing resources. The software’s capacity to process diverse image resolutions and scales makes it well-suited for identifying flaws in PV cells, since errors may vary greatly in both visual characteristics and size. In addition, the architecture of EfficientNet has been optimized to perform efficiently even when there is a scarcity of data, which is a typical obstacle in defect detection for PV systems. This is achieved by using sophisticated methods such as compound scaling [21].

EfficientNet is an excellent option when seeking innovative DL methods for detecting faults in PV cells using image-based techniques. Its exceptional combination of accuracy and efficiency makes it very remarkable. Table 1 provides a comparative study that explains the distinct skills and benefits of several DL models, such as ResNets (Residual Networks), CNNs, and others. EfficientNet stands out by using a methodical approach to increasing model size, resulting in improved performance without incurring unnecessary computational expenses. EfficientNet is well-suited for analyzing complex images of PV cells, since it excels at accurately detecting tiny abnormalities and flaws, which is crucial in this context. Several benchmarks and real-world applications outlined in the study further substantiate the choice to use EfficientNet in the research due to its established capacity to achieve exceptional accuracy in image classification tasks.

**Table 1:** Comparative analysis of DL algorithms for image processing.

Algorithm	Key Feature	Layers	Parameter Count	Primary Application	Accuracy/ Performance	Real-time Capability
**CNN**	Spatial hierarchy processing	Variable	High	Image classification	High	Moderate
**ResNet**	Deep networks with skip connections	Very Deep	Very High	Image classification	Very High	Low
**U-Net**	Symmetric expanding path	Deep	Moderate	Medical image segmentation	High	Low
**Visual Geometry Group (VGG)**	Deep with small convolution filters	Deep	Very High	Feature extraction	High	Low
**Inception Network**	Inception modules for multi-level features	Deep	High	Image classification	Very High	Moderate
**You Only Look Once (YOLO)**	Real-time object detection	Shallow	Moderate	Real-time object detection	Moderate	Very High
**Single Shot MultiBox Detector (SSD)**	Uses multiple feature maps	Moderate	High	Object detection	High	High
**Generative Adversarial Networks (GANs)**	Generator and discriminator networks	Deep	High	Image generation	–	Low
**Mask R-CNN**	Extends Faster R-CNN with egmentation	Very Deep	Very High	Instance segmentation	Very High	Moderate
**EfficientNet**	Scales depth, width, and resolution uniformly	Scalable	Moderate	Image classification	Very High	High

**Fig 2:**
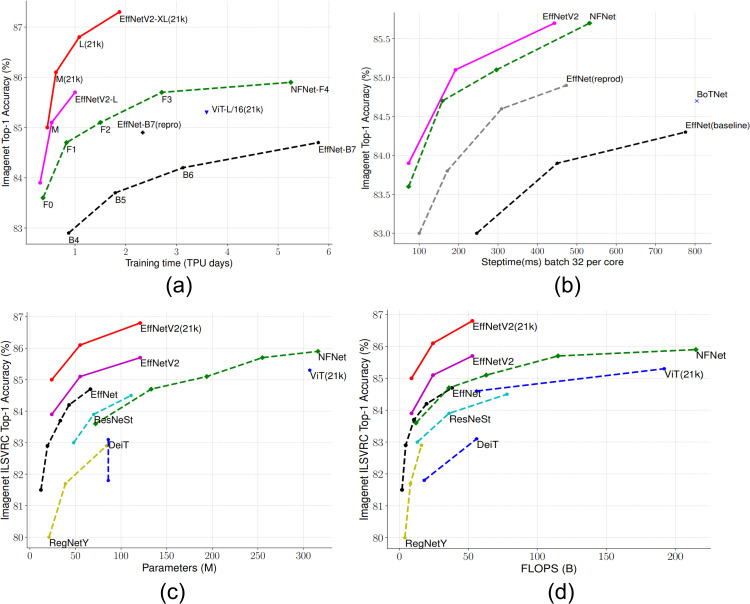
EfficientNetV2 Efficiency Comparison: (a) Training Time, (b) Step Time, (c) Parameters, and (d) FLOPs [22].

Fig 2 demonstrates the notable progress in both efficiency and efficacy of DL models during the research of EfficientNetV2. Fig 2(a) demonstrates the greater training efficiency of EfficientNetV2. It achieves higher accuracy on ImageNet ILSVRC2012 while using much fewer parameters and requiring less training time. This highlights its capacity for extensive implementation. In addition, Fig 2(b) illustrates the enhanced speed of the model during training iterations on TPUv3, emphasizing the decreased computational costs without any additional learning. Fig 2(c) demonstrates the model’s size efficiency, while Fig 2(d) illustrates the computational efficiency. These findings provide further proof of the superior performance of EfficientNetV2 compared to its earlier iterations [22]. Because of its exceptional characteristics, EfficientNetV2 is very suitable for detecting flaws in solar cells. The technology provides a significant degree of effectiveness and precision, which are essential for ensuring the long-term viability of energy-related applications.

Table 1 provides a comprehensive comparison of widely used Deep Learning (DL) models, clearly demonstrating EfficientNet’s balanced trade-off between accuracy, computational cost, and scalability—factors that are critical for high-performance and real-time fault detection in PV systems.

Fig 2 provides a multi-faceted comparison between EfficientNetV2 and other state-of-the-art models across key efficiency metrics—Fig 2(a) training efficiency, Fig 2(b) training step time, Fig 2(c) number of parameters, and Fig 2(d) FLOPs—showing that EfficientNetV2 achieves competitive ImageNet Top-1 accuracy with reduced training time and computational cost. These characteristics support its selection as the backbone of the proposed PV fault detection framework.

### 1.2. Motivation behind the study

The increasing worldwide transition to renewable energy sources highlights the crucial importance of PV systems in the contemporary energy environment [23]. As the use of solar energy increases, it is crucial to prioritize the operational efficiency of PV systems in order to maximize their energy production and, therefore, their economic sustainability and environmental advantages. Nevertheless, the functionality of these systems is sometimes hindered by several sorts of defects, such as microcracks, delamination, and soiling, which may greatly reduce their effectiveness.

Historically, the identification and assessment of these malfunctions have mainly depended on manual inspections and rudimentary automated techniques, which not only require a significant amount of work but are also susceptible to mistakes and discrepancies. Conventional approaches sometimes prove ineffective in identifying defects prior to causing significant deterioration to the solar panels [24]. Consequently, there is an urgent need for more sophisticated and dependable methods that may guarantee prompt identification and precise categorization of these defects in order to avoid significant harm and expensive periods of inactivity [25,26].

DL technologies provide a revolutionary option for automating and improving the problem detection process in PV systems. EfficientNet has gained prominence among several DL architectures for its exceptional capacity to handle visual input and scalability, resulting in excellent performance. The architecture’s efficient capacity to adapt its depth, breadth, and resolution makes it well-suited for effectively processing the complex and diverse images of PV cell defects [16,19,27].

In this research, the model proposes a complete DL framework for PV cells fault detection using EfficientNetV2 family. In contrast to previous studies focusing on a single model, this work conducts a comparative analysis of three distinct variants of EfficientNetV2—namely, EfficientNetV2B0, EfficientNetV2B2, and EfficientNetV2M—providing a detailed examination of the influence of model size on performance in PV fault detection tasks. The primary innovation is in the two-stage transfer learning approach utilized: first, the models undergo training in a feature extraction phase with base layers frozen, subsequently followed by a fine-tuning step in which upper layers are selectively unfrozen for domain-specific adaptation. This structured training method improves convergence and generalization. The model also includes the cell type (monocrystalline or polycrystalline) as an auxiliary input, therefore allowing a more knowledgeable and context-aware categorization procedure. Class weighting helps to balance data by guaranteeing the model stays sensitive to faulty samples, which are often under-represented. A very extensive image augmentation pipeline, in turn, enhances the model’s generalization capabilities to different real-life conditions. By using Grad-CAM to elucidate the regions responsible for triggering classification decisions, interpretability is ensured and the model’s behavior becomes transparent. Finally, the proposed approach is evaluated on a large-scale real-world EL PV dataset, illustrating its usefulness and building an apparent and scalable solution for intelligent PV fault detection with significant implications in the automated monitoring of solar farms and sustainable energy management.

This study proposes a DL method for automated fault detection in PV cells based on EfficientNetV2 architecture through EL imaging. This study presents a two-stage feature extraction based transfer learning strategy that brings in both frozen feature extraction as well as transfer learning focused towards fine-tuning, and facilitate the model to capture domain-specific patterns in a more efficient way. In contrast to conventional techniques, the framework uses class weighting to address data imbalance and integrates auxiliary cell-type information, guaranteeing more accurate and equitable classification across a variety of fault types. A more comprehensive data augmentation approach further supports the model’s generalization under various use-case scenarios. Finally, introducing Grad-CAM visualizations makes the decision-making process of the model transparent leading to interpretability and trust during real-world deployment. The proposed methodology comprehensively sets the path for sustainable fault diagnosis in modern energy systems with its scalability, interpretable, and rational predictions.

### 1.3. The problem statement

PV systems play a very important role in the renewable energy industry due to their ability to provide green and affordable energy but their effectiveness could be highly reduced because of various problems including microcracks, delamination and soiling of the PV surfaces. Such defects can significantly lower the electricity production and financial value of solar systems. Traditional methods to detect these defects are destructive and reliant on human inspection, or in simpler cases, basic automation which is less effective, costlier and subject to human error. Besides they are not complex enough to detect minor shortcomings which can translate into crucial bugs, if rectified not in timely manner [28–30].

Given the current limitations of technology, it is crucial to explore innovative approaches that can enhance the identification and assessment of issues in PV systems. EfficientNet, a type of architecture known for its exceptional image processing capabilities, has shown great potential, making DL a viable choice. EfficientNet is a highly regarded algorithm that is exceptionally skilled at processing complex visual data with great efficiency and effectiveness. This solution has the potential to revolutionize defect detection processes, offering a more precise, quick, and cost-effective alternative to current methodologies [31].

This research attempts to develop a DL model using the EfficientNet architecture to detect defects in PV cells and classify them. Through state-of-the-art ML techniques, this work establishes predictive maintenance approaches to enhance operational performance and lifetime of PV systems in solar energy. Instead, these advancements will naturally support the development of other sustainable solar energy solutions in the future without requiring long-term contributions, thereby promoting the continuous advancement of renewable energy technology.

Identification and diagnostic of PV system malfunctions have been traditionally reliant on various strategies, mainly based on current–voltage (I–V) characteristics analysis and ML methods. One of the most used approaches is a two-phase solution in which the parameters of a one-diode model are extracted first and then used with ML algorithms like Random Forest (RF) Classifiers, for anomaly detection and diagnosis. This approach has shown exceptional accuracy rates in classification assignments [32]. Other methods have used DL techniques, like deep residual networks, to automatically pull out features from raw I–V curves and environmental conditions. The above method has resulted in a significant improvement in the performance of defect identification, as shown in previous studies [33].

In addition, researchers have investigated the complete usage of I-V curves. This involves extracting features directly from the curves or using complex mathematical approaches to alter them. The modified curves are then examined using various ML classifiers to obtain a high level of diagnostic accuracy [34]. Advanced methods have been created to address more complex situations, such as numerous faults in PV arrays. These methods use multi-label learning to effectively manage the simultaneous occurrence of many faults [35]. Furthermore, the use of thermographic imaging in conjunction with CNNs has been employed to detect flaws by analyzing patterns of excessive heat in PV modules. This approach provides a non-invasive and effective diagnostic tool [36]. Furthermore, advanced models like dual-channel CNNs have been developed to improve the process of extracting and selecting features, hence maximizing the diagnosis of different fault states in the presence of complicated environmental factors [37].

Although PV systems must be included into the global energy matrix in order to be sustainable, these systems often experience faults that lower their efficiency. Advanced techniques for fault detection have been presented in recent work; Jalli et al. [38] created a DL model for reliable fault localization, while Hong and Pula [39] used a digital twin with a PSO-optimized transformer. Saravanan et al. [40] furthermore used a Binary Firefly Algorithm to reconfigure arrays in partially shaded conditions. Expanding upon these ideas, this study uses the EfficientNet architecture in our work to improve PV cell fault detection. In the end, this method tries to increase the stability and flexibility of fault detection under different circumstances, so promoting more reliable and effective solar energy systems.

These technologies provide a basic structure that guides the creation of more sophisticated systems such as EfficientNet for PV fault detection. These systems use more complex computational techniques to enhance accuracy and reliability in diagnosing faults, pushing the limits of what is possible.

### 1.4. The Objectives of the study

This study aims to design and validate a comprehensive DL architecture to efficiently and accurately detect various types of faults on PV cells using EL images. In particular, the work seeks to utilise the various architectures of the EfficientNetV2 architecture family to investigate how model depth and complexity relate to classification performance. To this end, three variants—EfficientNetV2B0, EfficientNetV2B2, and EfficientNetV2M—are implemented and systematically compared. This research further seeks to apply a two-stage transfer learning strategy comprising feature extraction followed by fine-tuning, with the goal of enhancing model generalization on PV fault detection tasks. The integration of auxiliary cell-type information (monocrystalline vs. polycrystalline) as a secondary input is also investigated to enrich the model’s contextual understanding and improve classification accuracy. Other goals relate to tackling class imbalance using weighted loss functions, utilizing various data augmentations for making the models more generalize, and also creating Grad-CAM visualizations to deliver transparency and interpret model predictions. In the end, the study aims to create a scalable, easy-explainable, and cost-effective DL pipeline ready for deployment in solar panel inspection and sustainable energy production monitoring systems.

## 2. Methodology

### 2.1. Data presentation

This study sourced data from a publicly accessible scientific research paper [41], providing extensive details on the efficiency and degradation of PV cells. This study used these datasets to identify and assess flaw patterns in PV cells, aiming to speed up fault detection through the use of EfficientNet-powered DL algorithms.

**Fig 3:**
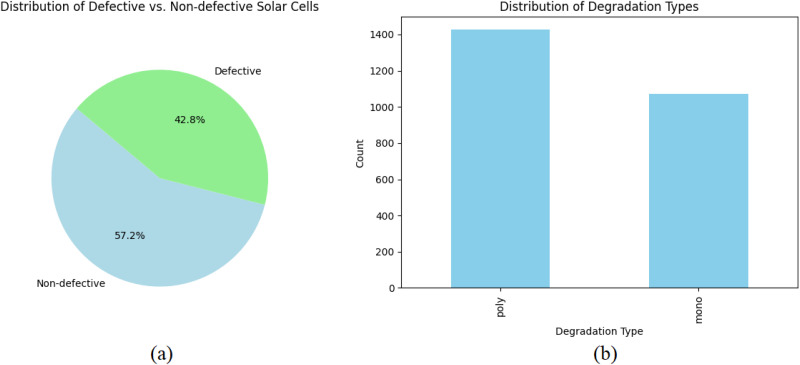
Analysis of PV Cell Conditions: (a) Proportion of Defective vs. Non-defective Cells, (b) Distribution of Cell Types (Monocrystalline vs. Polycrystalline).

Fig 3 presents a comprehensive visual examination of the information from two different perspectives. Fig 3(a) illustrates the distribution of faulty and non-defective cells, with a substantial number of cells being defective. This highlights the need for implementing efficient detection methods. Fig 3(b) shows the distribution of cell types. Acquiring this information is crucial for the development of a sophisticated DL model capable of precisely categorizing and forecasting various degradation patterns in PV cells.

Table 2 provides a comprehensive analysis of the defect rates documented in monocrystalline and polycrystalline cells. Based on the data, out of the total of 1071 faulty cells, 486 are monocrystalline, while 585 are polycrystalline. Out of the total of 1429 cells that are not defective, 588 are monocrystalline and 841 are polycrystalline. Among the defective cells, 45.38% may be attributed to malfunctions in monocrystalline cells, while the remaining 54.62% are a result of difficulties with polycrystalline cells. This data is essential for comprehending the distribution of defects and may suggest varying susceptibilities of cell types to certain aberrations.

**Table 2:** Comparative Analysis of Defectivity in Monocrystalline and Polycrystalline PV Cells.

Attribute	Defective	Non-defective
**Monocrystalline**	486	588
**Polycrystalline**	585	841
**Total**	1071	1429
**Monocrystalline (%)**	45.38%	41.15%
**Polycrystalline (%)**	54.62%	58.85%
**Total (%)**	100%	100%

Fig 3 and Table 2 provide a thorough examination of the occurrence and categorizations of flaws in PV cells, specifically emphasizing notable differences between Monocrystalline and Polycrystalline variations. This comprehensive investigation is crucial for customizing DL models to precisely identify and distinguish distinct categories of defects, hence enhancing the dependability and effectiveness of PV cells.

Properly splitting the dataset into training, validation, and test subsets is an important part of developing a strong ML model. In this study, the original dataset of 2,500 EL images of PV cells were divided into training (80% of the dataset), validation (10% of the dataset) and testing (10% of the dataset) sets using a stratified sampling approach to ensure the class distribution across all the subsets. This study implemented this using a fixed random seed with the ‘Scikit-learn’ ‘train_test_split()’ function, maintaining reproducibility and avoiding data leakage.

The original EL images are 300 × 300 pixels (grayscale), converted to 3-channel RGB, and then resized to 224 × 224 × 3 before being fed into the EfficientNetV2 models. Pixel values range in [0, 255] were divided by 255 to normalize to [0,1] so the data has similar scales and allows the network to converge better while training.

Furthermore, ‘TensorFlow’s’ ‘ImageDataGenerator’ was used to set apply a set of data augmentation (rotation (±30°), zoom (±20%), shearing, horizontal and vertical flipping) just to training data. This augmentation pipeline greatly enhanced the model’s capacity to generalize to in-field deviations in PV cell degradation. On the other hand, the validation and test datasets weren’t augmented and only rescaled, as they should reflect how the model performs in realistic conditions where the model sees new data.

This modified partitioning strategy enabled fair comparison across the three tested models (EfficientNetV2B0, B2, and M) while improving training stability and allowing a clean, held-out evaluation metric from the test set. The final distribution of the dataset is outlined in Table 3.

**Table 3:** Distribution of PV cell image data for training, validation and test sets.

Set	Image Format	Number of samples/images	Percentage
**Training set**	300 x 300 x 3 (RGB)	2000	80%
**Validation set**	300 x 300 x 3 (RGB)	250	10%
**Test set**	300 x 300 x 3 (RGB)	250	10%

**Fig 4:**
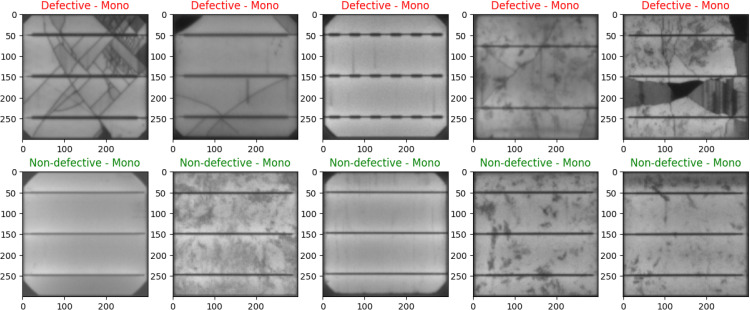
Comparative Display of Defective and Non-Defective Monocrystalline PV Cells.

**Fig 5:**
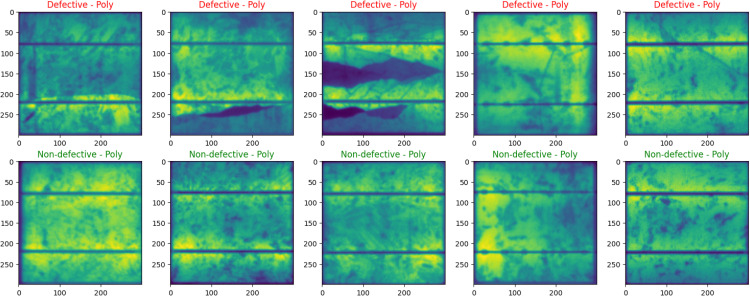
Comparative Display of Defective and Non-Defective Polycrystalline PV Cells.

Within the dataset presentation component of our study, this study does a comprehensive visual analysis to differentiate between defective and operational PV cells, with a particular emphasis on their physical attributes. Fig 4 and Fig 5 are essential in this visual analysis as they provide a meaningful comparison via the presentation of a comprehensive range of images. Fig 4 mainly showcases monocrystalline cells, including a sequence of images illustrating both flawed and flawless instances. This section is crucial for emphasizing the visual differences that differentiate faulty cells from non-defective cells, thereby establishing a solid foundation for comprehending possible losses in efficiency in monocrystalline solar systems. Fig 5 employs a comparison methodology to analyze polycrystalline PV cells. It showcases a balanced collection of 5 faulty cells and 5 non-defective cells. Comparing these materials is crucial in order to fully understand the distinct defect patterns that are characteristic of polycrystalline materials, which enhances our visual analysis of the dataset.

The images shown in Fig 4 and Fig 5 only reflect a tiny portion of a whole dataset, including a total of 2,500 images. The purpose of including these images was to visually depict the differences in appearance between monocrystalline and polycrystalline cell types, as well as to highlight the changes in appearance between defective and non-defective states. By directly comparing the cells, our goal is to clarify the unique features and flaws that are naturally present in both kinds. By doing this, this study provides a strong basis for our future study, which aims to improve the procedures of identifying and categorizing faults in these crucial components for solar energy systems.

### 2.2. Modeling and analysis of PV cell defects

#### 2.2.1. Model setup and data preprocessing.

The research starts by setting up the required Python environment and importing important libraries such as ‘pandas’, ‘numpy’, ‘matplotlib’, and ‘TensorFlow’. These tools are essential for manipulating data, doing numerical calculations, creating visualizations, and conducting DL operations. The first step entails establishing the work environment, beginning with the installation of Pillow—a robust library for image processing that enables efficient handling and modification of PV cell images. Next, the working directory is assigned to a designated folder, which is carefully organized to efficiently manage all pertinent code and data.

To proceed, the following step involves using a specialized utility function called ‘elpv_reader.load_dataset’ to import the dataset. This dataset comprises images of PV cells, together with their corresponding defect probabilities and cell kinds. In order to preprocess the data for ML methods, this study transforms the textual labels representing cell kinds, such as monocrystalline and polycrystalline, into numerical labels. In addition, this study converts the probabilities that indicate the possibility of flaws into binary classifications, making them obvious and distinct: faulty (1) or not defective (0). Given that the model requires RGB inputs, but the given images are in grayscale, this study addresses this issue by converting the images to RGB format. This is accomplished by replicating the grayscale channel three times along the color channel dimension.

The dataset is partitioned into training and validation sets using the ‘train_test_split’ function from the ‘scikit-learn’ library, which guarantees a systematic approach to training the model and evaluating its performance. In addition, this study performs a comprehensive analysis of the data’s properties, which involves computing and presenting the full range of pixel intensities. This enables us to enhance our comprehension of the variability of the data. Moreover, this study analyzes the ratio of faulty and non-faulty cells to examine the distribution of categories and detect any possible prejudices.

**Fig 6:**
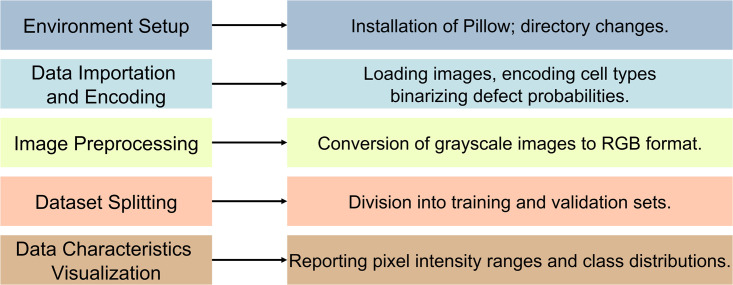
Summary of Data Preparation and Model Configuration Parameters.

The meticulous preparation procedures and well-structured configuration, as seen in Fig 6, are crucial for the successful deployment of a robust DL model. By using TensorFlow’s Keras Application Programming Interface (API) and utilizing advanced topologies like EfficientNetV2, this study improves the prediction capabilities of our model. This complete configuration not only readies the dataset for efficient training, but also establishes the conditions for thorough assessment and validation of the model’s proficiency in identifying flaws in PV cells.

#### 2.2.2. Data augmentation strategies.

**Fig 7:**
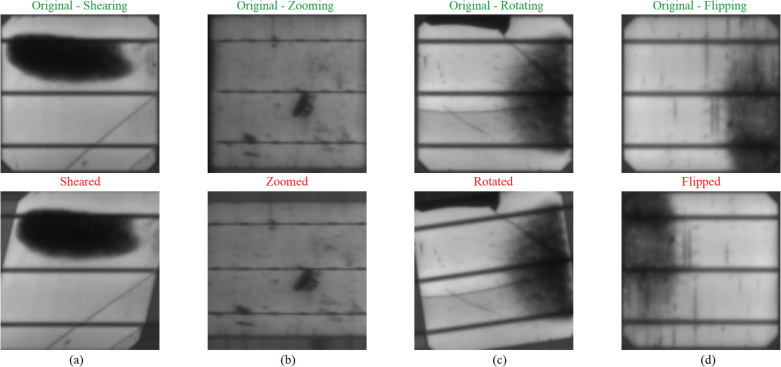
Visualization of Data Augmentation Techniques on PV Cells(a) Shearing, (b) Zooming, (c) Rotation, (d) Flipping.

When preparing our model for recognizing faults in PV cells, this study put a lot of weight on data augmentation, as it helps to enhance the generalization abilities of the model. The training images are dynamically augmented using TensorFlow’s Keras preprocessing tools. For example, this study shear, zoom, rotate and flip them as shown in Fig 7. Shearing is done at certain angles, which transforms the images so that the model can identify cells from different viewpoints. Thereby enabling the model even to know about defects on parts of its original image having different sizes but same details by zooming into those areas while rotating it up to 30 degrees and flipping both horizontally and vertically allows for understanding defects observed in various orientations or mirror images respectively. These changes create diverse situations within a strong dataset which is important because it exposes many examples to learn from thus increasing performance when dealing with unseen data. Additionally, the dataset is systematically partitioned into training, validation, and test sets using the ‘train_test_split’ function from ‘Scikit-learn’, with 80% allocated for training, and the remaining 20% equally divided between validation and testing. This systematic division ensures that the model will be evaluated on blind data, providing an honest and realistic estimation of the ability to generalize. This study prepares the training data using a combination of image augmentation techniques such as rotation, zoom, shear and flip, thereby making the model more robust to variations. In addition, all pixel values in the image are scaled to [0, 1] by dividing them by 255 to have a consistent scale and help with faster convergence during training. The validation and test datasets, on the other hand, are only rescaled (not augmented) to mimic real-world inference conditions as closely as possible. The identity of generators to create data performed training are not only obtained from the varied and normalized input, but the identity of generators for evaluation also represent the model’s ability to distinguish defects from PV cells under real operating conditions.

#### 2.2.3. Transfer learning with EfficientNetV2 models.

In this study, it uses three versions of EfficientNetV2 architecture: EfficientNetV2B0, EfficientNetV2B2, and EfficientNetV2M, with pre-training on the large and diverse ImageNet dataset to take advantage of transfer learning. This technique offers our models a strong ability to extract features learnt from a variety of natural images, which improves baseline performance, and accelerates convergence in comparison to if it were to learn from scratch.

The EfficientNetV2 family utilizes a compound scaling method which fine-tunes the balance between depth, width and resolution in an accurate, consistent manner to provide state-of-the-art accuracy and computational efficiency. This characteristic allows its use in both, the detection of faults in PV cells, that requires precision but also efficiency.

This study does not use these models in their default forms. Instead, each model is integrated into a two-stage transfer learning strategy, where the base model is first used as a fixed feature extractor, followed by selective fine-tuning of the upper layers using our domain-specific dataset comprising PV cell EL images. This fine-tuning process helps the model focus more closely on task-relevant features—such as micro-cracks or hotspots—that indicate cell degradation or malfunction.

In addition, it has batch normalization and dropout layers in the architecture, which are indispensable during training. Making network less sensitive to the initial learning rate and also helps learn faster (stabilizing the training process) by normalizing the input to each layer. Dropout refers to a regularization technique that randomly disables units during training − preventing overfitting and helping the network to learn more generalized representations of features.

Using several EfficientNetV2 variants this enables a comparison of performance from simple models with low capacity up to high capacity model. This not only guarantees an optimized architecture for PV fault detection enables insights on the scalability and deployability for disparate hardware specifications. With a powerful model architecture, transfer learning with pre-trained weights and fine-tuning, our framework proves capable of adapting to the high variability present in PV cell images. This boils down to enabling highly accurate, reliable, and explainable detection of faults, which will help give rise to smarter, more efficient solar energy systems as well as further sustainability efforts within the renewable energy industry.

The implementation code for the EfficientNetV2B0, EfficientNetV2B2, and EfficientNetV2M experiments is provided in the Supporting Information (S1 File).

#### 2.2.4. Training execution.

The model training is specifically planned to give great results over several epochs. The TensorFlow had been embedded in the model to dynamically adjust the learning rate in real-time, which significantly boosts the efficiency of the model learning. A custom callback (‘DecreaseLR’) was used to reduce the learning rate by a factor of 3 every 2 epochs after epoch 4. By doing this, the weights in the network are effectively adapted over the course of training, so that the model can avoid getting trapped in local minima too early on.

Additionally, it uses checkpointing with the ‘ModelCheckpoint’ function to save the best model according to its validation set performance. It can be done by monitoring specifically the ‘val_categorical_accuracy’ metric. It only saves the best version of the model based on how well it does with new, unseen data. This method reduces over-fitting, keeping the model more resilient in different environments.

The training dataset utilized in this research, is enhanced using ‘ImageDataGenerator’ method, that is used to apply transformation such as rotation, zoom, shear and flips to amount better variety of input. These augmentations offer a wider visual viewpoint that aids in bridging the gap between the training data and actual world. A full training pipeline is run separately for each of the three models, EfficientNetV2B0, EfficientNetV2B2 and EfficientNetV2M, which serves to structure a performance comparison over architectures with different complexity. Both models are trained in two steps: then in another phase, the top layers are unfrozen in to adjust these to the patterns of the PV cell image data specifically. This aggressive training approach is largely designed to provide the models with a diversity of possible input conditions that best match the expansive variability observed in real-life solar cell scenarios.

#### 2.2.5. Model evaluation.

Model performance is assessed using plots for accuracy and loss over the epochs at the end of training. Training and validation Mode—These metrics are plotted for a clear picture of the learning trajectory. Matplotlib is used to create charts for training vs validation accuracy in one subplot and training vs validation loss in the other.

These graphical structures are important because they help determine the success of model learning. They also allow for identification of whether the model is overfitting or underfitting, resulting in insufficient learning. The curve on the accuracy plot helps us explore how close the model is when predicting the correct category over time, and comparing it to the curve on the loss plot gives us an insight into how the model minimizes the loss function.

Finally, this study looks at Precision, Recall, and F1-Score as a set of classification performance metrics along with Accuracy and Loss to evaluate the models. These metrics give a more balanced perspective on how the model behaves with imbalanced datasets like the dataset presented in this research, where the classes defective cells are less than the non-defective ones. Precision is about what you predict as defective, how many are actually defective, Recall shows what percent of the actual defective were predicted as defective, and the F1-Score balance the both to provide a unified performance metric.

To further assess the classification behavior, a confusion matrix is plotted, which shows the true positives, false positives, true negatives, and false negatives. This matrix gives a visual representation of how well the model distinguishes between the two classes.

This study uses the mentioned metrics for serious assessment, ensuring empirical proof of the model-based solution’s effectiveness through statistical evidence. Through this step-by-step training and evaluation routine, reinforced by our dedication to using just those EfficientNetV2-based models (B0, B2, and M) that have endured rigorous tests and locked-out errors as our favourite candidates for identifying any defects in the PV cells, this study aims to ensure that only the best quality models are deployed.

#### 2.2.6. GradCAM visualization in model predictions.

A crucial visualization method used in our study is Gradient-weighted Class Activation Mapping (Grad-CAM). It gives us key insights of how our neural network has decided and why. This method is important to evaluate the prediction of the model for PV cells. Specifically, the process entails tracking the gradients of the desired output (such as a class label indicating a fault) as it passes through the last convolutional layers of our EfficientNetV2 models.

**Fig 8:**
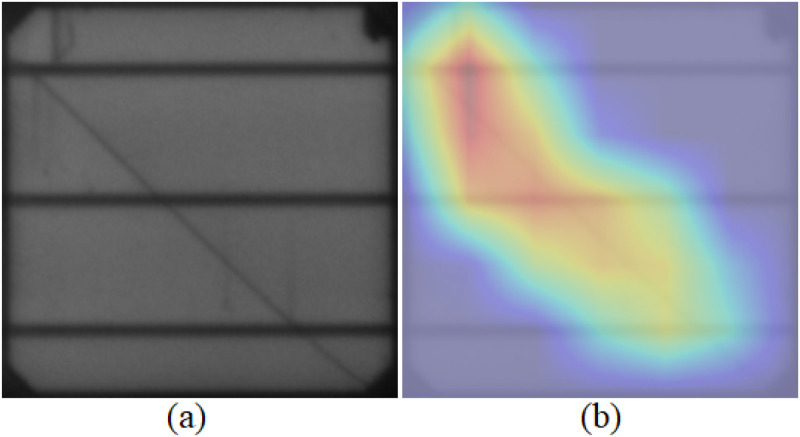
Comparison of Original and GradCAM-Enhanced PV Cell Images(a) Original Image, (b) GradCAM Enhancement.

Actually, the red areas in the images indicate the areas that really influence the model’s predictions, according to Grad-CAM. That suggests age-related issues like cracks and other flaws. Additionally, the Artificial Intelligence (AI) system uses a model that enhances its accuracy and stability as a robust diagnostic tool. If the heatmaps repeatedly draw more attention to unimportant features, this can be evidence of the need for models for better data preparation or training. Using the Grad-CAM algorithm encourages the model’s performances to be more transparent and builds trust in the model’s skills around the solar cell’s features, which leads to solar cell anomalies. This robust interpretability method plays a major role in helping us gain a deep understanding of and confidence in the model’s ability to discern the inner patterns of the data. Such information will greatly assist clients in their decision-making process regarding the maintenance and management of solar panels. Fig 8 provides a visual representation of the application of Grad-CAM in the research.

The process begins by recording the activations of the feature map $A^{k}$ in the last convolutional layer, namely from the k−th channel. These activations are essential for catching the more advanced information from the input image. The activations are next used to calculate the gradients of the output class, represented as $y^{c}$, in relation to the feature maps. This effectively reveals the extent to which each feature map contributes to the output class score, as shown by Equation (1).

Furthermore, these gradients also undergo another operation which is the Global Average Pooling (GAP) to compute the neuron importance weights, $\overset{c}{\underset{k}{\propto }}$ that determines the overall importance of each channel with respect to class c, defined as the Equation (2). The process is simply taking the mean of the gradients across all the spatial i , j locations within each map. The procedure’s final step is obtaining the weighted sum of the forward activation maps and applying the Rectified Linear Unit (ReLU) function to generate the class-discriminative localization map indicated as $\;\overset{c}{\underset{Grad-CAM}{L}}$.

The shown map delineates the precise locations that have a favorable influence on the decision-making process of the model, thereby delineating the pivotal regions for the predictions generated by Equation (3).

**Fig 9:**
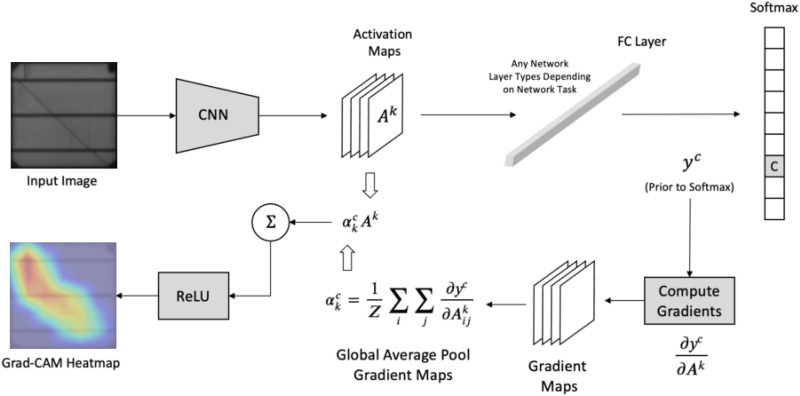
Grad-CAM Process Flowchart: From Input to Heatmap Visualization.


To see a formal graphic representation of this process, refer to Fig 9. The supplied diagram shows the entire Grad-CAM process from the input image progressing through multiple network layers to the production of the heatmap.

$$\frac{\partial y^{c}}{\partial A^{k}}$$ (1)

$$\begin{array}{c} \overset{c}{\underset{k}{\propto }}=\frac{\text{1}}{Z}\sum {\mathrm{\backslash nolimits}}_{i}\sum {\mathrm{\backslash nolimits}}_{j}\frac{\partial y^{c}}{\partial \overset{k}{\underset{ij}{A}}}\; \end{array}$$ (2)

$$\begin{array}{c} \overset{c}{\underset{Grad-CAM}{L}}=\text{ReLU }(\sum {\mathrm{\backslash nolimits}}_{k}\overset{c}{\underset{k}{\propto }}A^{k}) \end{array}$$ (3)

Table 4 provides a comprehensive and convenient summary of key configuration factors and settings for the PV cell fault detection model. Therefore, in this study provides valuable information about image processing changes, data augmentation methods, and architecture programming of the DL model. It is important to include Table 4, for it is the essential method of this study. It presents a detailed explanation of how this work specifically created the dataset, designed the model architecture based on EfficientNetV2 architectures, and the training parameters that were optimized including the batch size and learning rate schedules. Hence, through Table 4, one would have an insight into the key features which help the model learn from PV cells correctly and predict defects correctly as well.

**Table 4:** Key Configuration Parameters and Settings for PV Cell Defect Detection Model.

Parameter	Description
**Library Imports**	Pandas, NumPy, Matplotlib, TensorFlow, Scikit-Learn
**Dataset**	Loaded using a custom function; includes EL images, defect probabilities, and cell type labels
**Image Conversion**	Grayscale converted to RGB to match EfficientNetV2 input requirements
**Data Splitting**	80% Training, 10% Validation, 10% Testing
**Batch Size**	32
**Image Size**	Resized to 224 × 224
**Data Augmentation**	Shear (0.1), Zoom (0.2), Rotation (30°), Horizontal and Vertical Flip
**Base Models Used**	EfficientNetV2B0, EfficientNetV2B2, EfficientNetV2M (all pre-trained on ImageNet)
**Fine Tuning**	Optional; specific layers unfrozen after initial training.
**Learning Rate**	Initially set to 0.01 for Stage 1; reduced to 1e-4 for fine-tuning (Stage 2)
**Epochs**	Stage 1: 50 epochs (Feature Extraction), Stage 2: 50 epochs (fine-tuning)
**Model Output**	2-class softmax (defective vs. non-defective)
**Performance Metric**	Categorical Accuracy
**Model Checkpoints**	Best-performing models saved based on validation accuracy
**Class Weights**	Manually defined: {0: 0.5, 1: 1.5} to address class imbalance
**Explainability**	Grad-CAM/ Score-CAM applied for defect localization and model interpretation
**Image Normalization**	Pixel values rescaled to [0, 1] using rescale = 1./255
**Input Channels**	3 (converted to RGB from grayscale)
**Activation Function**	Softmax used in output layer for binary classification
**Loss Function**	CategoricalCrossentropy(from_logits = False)
**Dropout Rate**	0.2 (applied after GAP)
**Normalization Layers**	BatchNormalization added before and after pooling layers
**Learning Rate Scheduler**	Custom callback reduces learning rate by factor of 3 every 2 epochs after epoch 4
**Optimizer**	Adam optimizer used in both training stages
**Evaluation Metrics**	Accuracy, Loss, Precision, Recall, F1-Score, Confusion Matrix
**Test-Time Evaluation**	Conducted using best checkpoint model on unaugmented test set
**Random Seed**	Set to 64 for reproducibility

### 2.3. EfficientNetV2 resource analysis

Present in Table 5 a comparison of the number of parameters, memory consumption, and computation complexity for the three EfficientNetV2 variants used in this study (B0, B2 and M). Naturally, EfficientNetV2B0 is the lightest model – the least number of parameters and smallest memory footprint, making it a viable solution for deployment on low-resource devices. EfficientNetV2B2 provides a good balance of efficiency and accuracy, while EfficientNetV2M, with the highest complexity, requires three times the computational power and may lead to improved accuracy as a by-product of more depth and parameter capacity.

**Table 5:** Comparison of Model Size, Memory Requirements, and Computational Complexity Across EfficientNetV2 Variants.

Model	Number of Parameters (Approximate)	Memory Usage (Approximate)	Computational Complexity (Approximate)
EfficientNetV2B0	7.1 - 7.2 Million	29 MB	Lower
EfficientNetV2B2	10.1 - 10.2 Million	40 MB	Medium
EfficientNetV2M	54.1 - 54.4 Million	227 MB	Higher

Table 5 compares the three EfficientNetV2 variants based on their model size, memory use, and computational complexity. This information can help researchers choosing the best model for their real-world PV monitoring needs based on the limitations of their application and the hardware available.

### 2.4. Evaluation metrics

When assessing our PV cell defect detection model, this study mainly uses two metrics: categorical accuracy and categorical cross entropy loss. It takes just dividing the number of accurate predictions by the total number of predictions made to get the Categorical Accuracy Equation (4). In especially helpful for tasks requiring classification, this simple formula offers a clear assessment of accuracy.


$$Categorical\;Accuracy=\;\frac{Number\;of\;Correct\;Predictions}{Total\;Number\;of\;Predictions}$$ (4)

Conversely, the Categorical Cross-Entropy Loss, as presented in Equation (5), evaluates the predicted probability distributions against the actual label distributions to measure the divergence from the true outcomes. This loss function serves as a critical indicator of how well the model’s probabilistic predictions align with reality, quantifying the extent of prediction inaccuracy in terms of probability.

Where M in Equation (5) is the number of classes,$\;y_{o,c}\;$is a binary indicator (0 or 1) if class label c is the correct classification for observation o, and $p_{o,c}\;$is the predicted probability observation o is of class c.


$$\begin{array}{c} Categorical\;Cross\;Entropy=\;-\sum \overset{M}{\underset{c=\text{1}}{\mathrm{\backslash nolimits}}}y_{o,c}\text{log}(p_{o,c}) \end{array}$$ (5)

Besides these basic metrics, this research also includes other main classification metrics as Precision, Recall, F1-Score, and the Confusion matrix. Precision measures the percentage of positive cases predicted correctly, out of the total number of positive predictions produced by the model, reducing false positives and is formulated as in Equation (6). Recall reflects how well the model correctly identifies all real positive cases, with the goal of reducing false negatives, and is defined in Equation (7). The F1-Score is the harmonic mean of Precision and Recall, so it captures a balanced measure of both dimensions, especially beneficial in datasets where class distribution is imbalanced like the dataset in this paper, and it is calculated as shown in Equation (8).


$$Precision=\;\frac{True\;Positives}{True\;Positives\;+\;False\;Positives\;}$$ (6)

$$Recall=\;\frac{True\;Positives}{True\;Positives\;+\;False\;Negatives\;}$$ (7)

$$F\text{1}-Score=\;\text{2}\times \frac{Precision\;\times \;Recall}{\;Precision\;+\;Recall\;}$$ (8)

The quantitative evaluation can be complemented by the confusion matrix which allows observing in detail the behavior of the model regarding true positives, false positives, true negatives, and false negatives. These other metrics give a more detailed and comprehensive measure of performance than accuracy alone. These metrics collectively provide a good overview of performance. The data shapes the training and fine-tuning of the models to provide predictions that are not only in terms of accuracy but also appropriate probabilistic representations to be utilized in the practical deployment of rest and PV cells.

## 3. Results and discussion

The performance of the three selected EfficientNetV2 models for pv cell fault detection is shown in Table 6. The best architecture for the evaluated models, EfficientNetV2M, excels among the evaluated models on all metrics achieving the highest accuracy (0.896), precision (0.886), recall (0.775) and F1-score (0.827). These results show that this study can better recognize faults correctively while keeping a good trade-off between precision and recall. EfficientNetV2B2 achieved accuracy of 0.832 shows a moderate achievement with no more than a minimal improvement towards the results produced by EfficientNetV2B0. On the other side, EfficientNetV2B0 lags significantly behind, for example in recall (0.563), which indicates that it is not as good at detecting all relevant fault cases. In summary, the best model for reliable and efficient detection of faults in PV cells is without a doubt EfficientNetV2M as outlined clearly in Table 6.

**Table 6:** Comparative Performance Metrics of EfficientNetV2 Models for Photovoltaic Cell Fault Detection.

Model	Accuracy	Precision	Recall	F1-Score
**EfficientNetV2B0**	0.820	0.818	0.563	0.667
**EfficientNetV2B2**	0.832	0.806	0.625	0.704
**EfficientNetV2M**	**0.896**	**0.886**	**0.775**	**0.827**

EfficientNetV2M delivered the best results since faults in PV cells in EL images, including microcracks and early discoloration, are often small and localized, which requires a strong fine-detailed feature extraction with sufficient global context, which prevents the misclassification of defect patterns. The higher capacity of V2M, with extra layers and channels, compared to B0 and B2, allows the preservation and combination of weak texture cues at many scales, which improves defective-sample detection, minimizes false negatives, and is consistent with its high recall and F1-score. Overall, the model can describe both small receptive-field information, like thin cracks, and large contextual information, such as distributed degradation. This results in more dependable classification.

The training and validation performance of EfficientNetV2 models during the feature extraction phase is shown in Table 7. With a best validation accuracy of 0.824 obtained at epoch 10, EfficientNetV2B2 exhibits rapid convergence to optimal performance. Moreover, this model achieved an almost uniformly good training-to-validation performance (the training accuracy was 0.725, and the validation loss was the lowest at 0.450), but it took 28 total epochs—again, stable learning over a long training period. Although EfficientNetV2M reached the highest training accuracy (0.732) and relatively competitive validation loss (0.455) score, its best validation accuracy was limited to 0.808. This indicates that while EfficientNetV2M was successful at fitting the training data, it may have had some slight generalization issues in this phase. EfficientNetV2B0, albeit attaining optimal performance at epoch 13, achieved the lowest performance among the three with a best validation accuracy of 0.816 and lowest training accuracy (0.707). As detailed in Table 7, these results emphasize the feature extraction stage of EfficientNetV2B2 as the overall best-performing one, considering the trade-off between accuracy and loss over epochs.

**Table 7:** Comparative Training and Validation Performance of EfficientNetV2 Models During Feature Extraction Stage.

Model	Best Validation Accuracy	Epoch of Best Validation Accuracy	Training Accuracy (Best Epoch)	Training Loss (Best Epoch)	Validation Loss (Best Epoch)	Total Epochs Run
**EfficientNetV2B0**	0.816	13	0.707	0.497	0.517	15
**EfficientNetV2B2**	0.824	10	0.725	0.561	0.450	28
**EfficientNetV2M**	0.808	10	0.732	0.482	0.455	20

Table 8 displays the training and validation metrics of the EfficientNetV2 models at the epoch corresponding to the lowest validation loss throughout and first stage (feature extraction). In addition to achieving the lowest validation loss of 0.408, the highest training accuracy of 0.744 was also achieved by EfficientNetV2M, suggesting the highest learning capacity and efficient feature representation among architectures. Nonetheless, it exhibits the lowest validation accuracy (0.796) compared to all the models. The EfficientNetV2B2 model attained optimal generalization performance, with a maximum validation accuracy of 0.816 and a minimum validation loss of 0.426. It attained this ideal condition at epoch 23, signifying that further training enhanced its generalization capabilities. At epoch 10, EfficientNetV2B0 had a validation accuracy of 0.808 and a validation loss of 0.480, indicating that the model efficiently learned features but only moderately generalized well to the validation data. EfficientNetV2M was the most efficient in terms of loss minimization, and EfficientNetV2B2 was the better overall validation accuracy as shown in Table 8, demonstrating a trade-off between training efficiency and generalization performance.

**Table 8:** First stage – (Feature Extraction) Training and Validation Metrics at Best Epoch Based on Minimum Validation Loss for EfficientNetV2 Models.

Model	epoch	Training Accuracy	Training Loss	Validation Accuracy	Validation Loss
**EfficientNetV2B0**	10	0.707	0.527	0.808	0.480
**EfficientNetV2B2**	23	0.711	0.483	0.816	0.426
**EfficientNetV2M**	15	0.744	0.430	0.796	0.408

Table 9 shows the training and validation performance of the EfficientNetV2 models in second stage (fine-tuning) of the experiment, measured at the epoch at which each model achieved its lowest validation loss. The fine-tuning method revealed that EfficientNetV2M outperformed other models in the steadfastness, validity, and suitability for diagnosis, both in terms of training accuracy (0.830), validation accuracy (0.844), training loss (0.289), and validation loss (0.304). As these results show, EfficientNetV2M not only learned well from the training set, but generalized incredibly well on the validation set. EfficientNetV2B0 was right behind with a validation accuracy of 0.820 and a light higher validation loss of 0.429, demonstrating stable generalization after training for 23 epochs. Although EfficientNetV2B2 achieves relatively the same training accuracy (0.741), the validation accuracy and validation loss were lower being 0.800 and 0.438 respectively. In general, the performance in the fine-tuning phase shows that out of all, the EfficientNetV2M alone proves to be the strongest model both regarding learning efficiency as well as generalization ability.

**Table 9:** Second Stage – (Fine Tuning) Training and Validation Metrics at Best Epoch Based on Minimum Validation Loss for EfficientNetV2 Models.

Model	Epoch at Minimum Validation Loss	Training Accuracy	Training Loss	Validation Accuracy	Validation Loss
**EfficientNetV2B0**	23	0.739	0.425	0.820	0.429
**EfficientNetV2B2**	16	0.741	0.427	0.800	0.438
**EfficientNetV2M**	12	0.830	0.289	0.844	0.304

**Fig 10:**
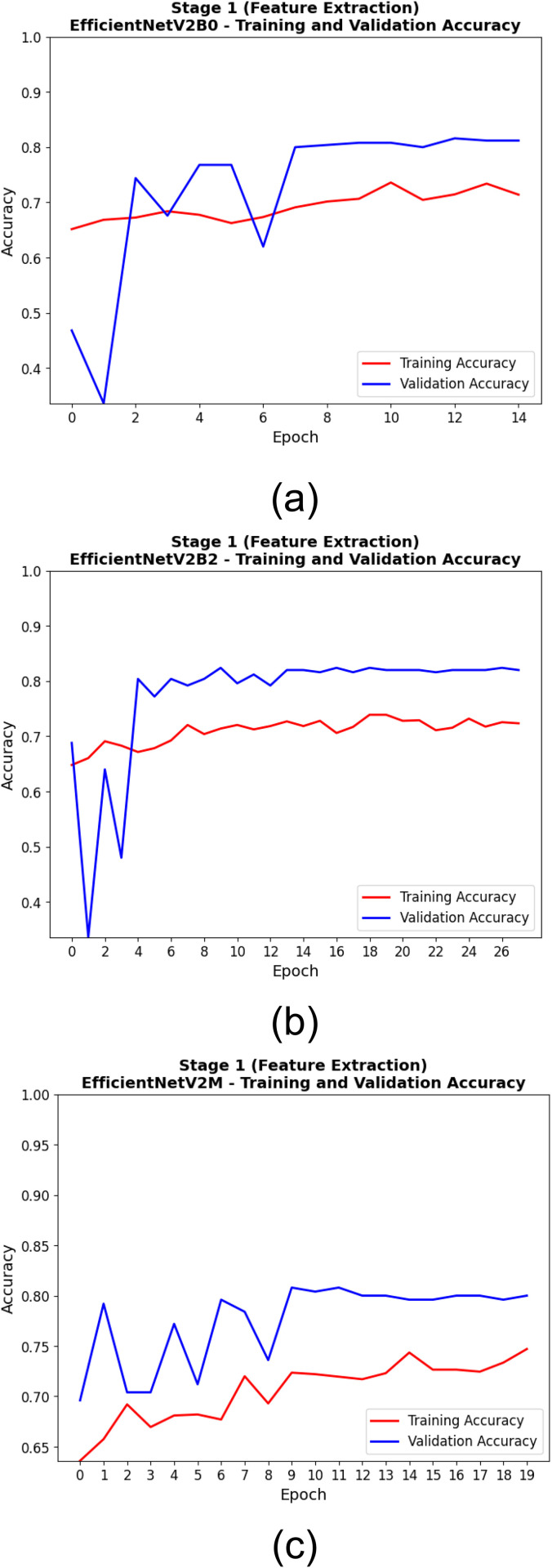
Training and Validation Accuracy Curves During the First Stage (Feature Extraction) for (a) EfficientNetV2B0, (b) EfficientNetV2B2, and (c) EfficientNetV2M Models.

**Fig 11:**
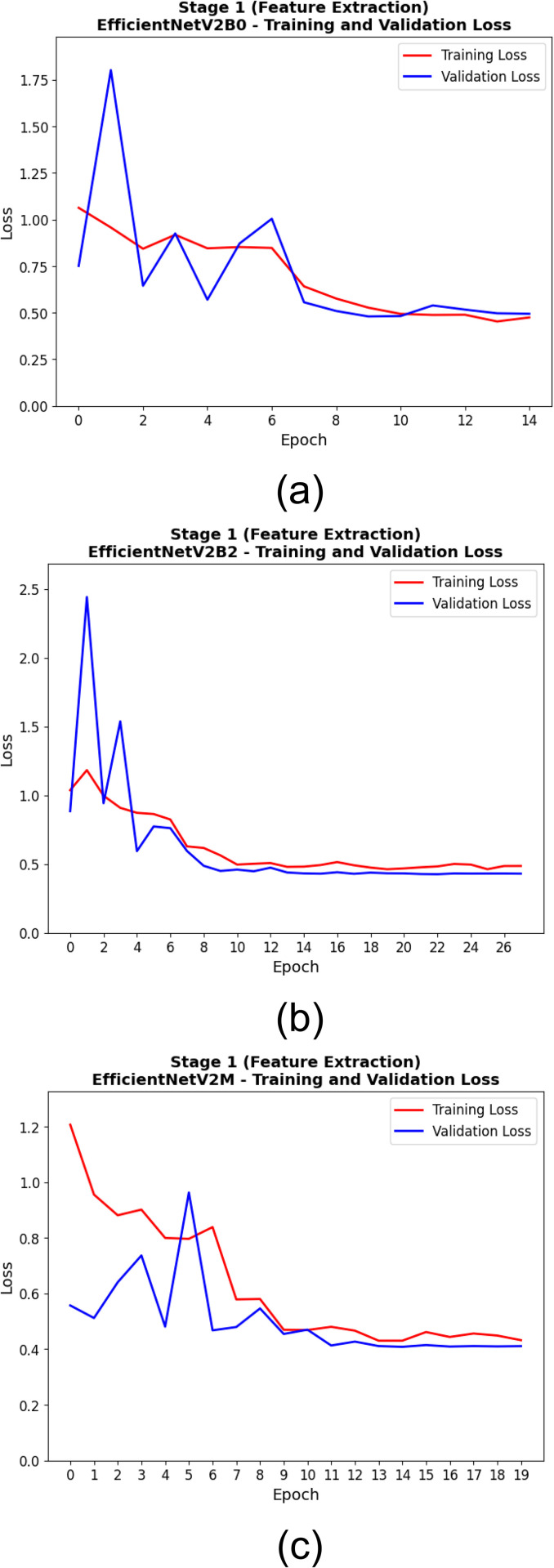
Training and Validation Loss Curves During the First Stage (Feature Extraction) for (a) EfficientNetV2B0, (b) EfficientNetV2B2, and (c) EfficientNetV2M Models.

**Fig 12:**
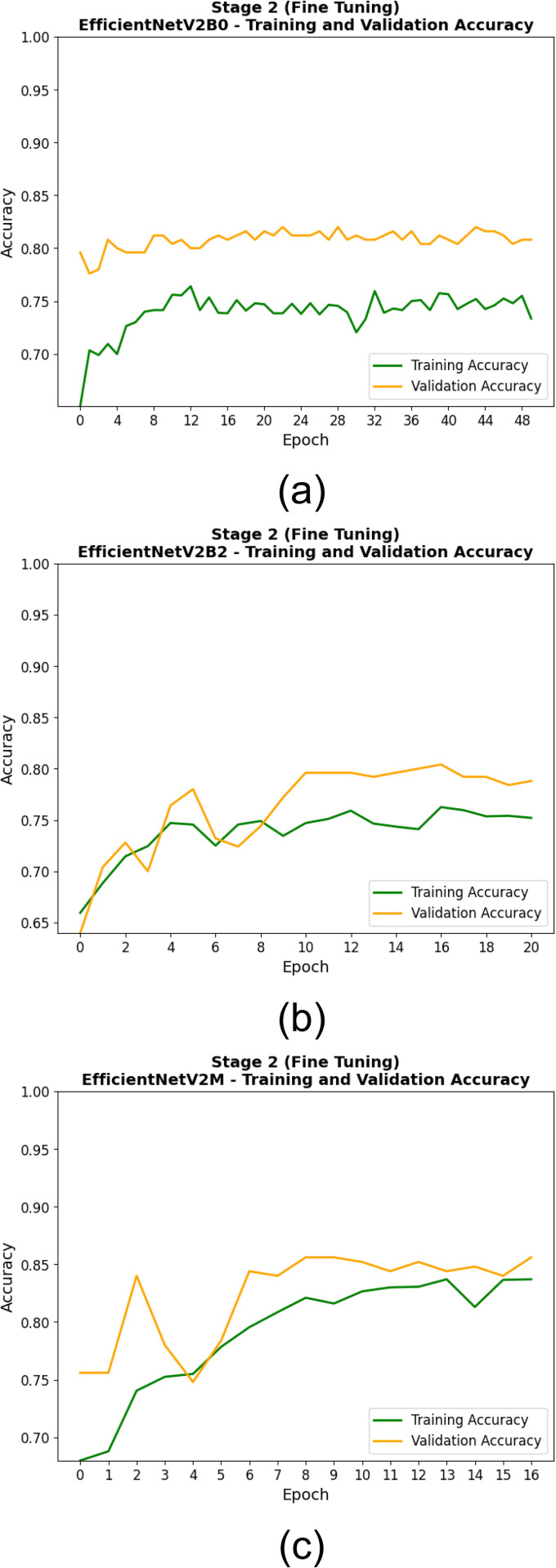
Training and Validation Accuracy Curves During the Second Stage (Fine-Tuning) for (a) EfficientNetV2B0, (b) EfficientNetV2B2, and (c) EfficientNetV2M Models.

**Fig 13:**
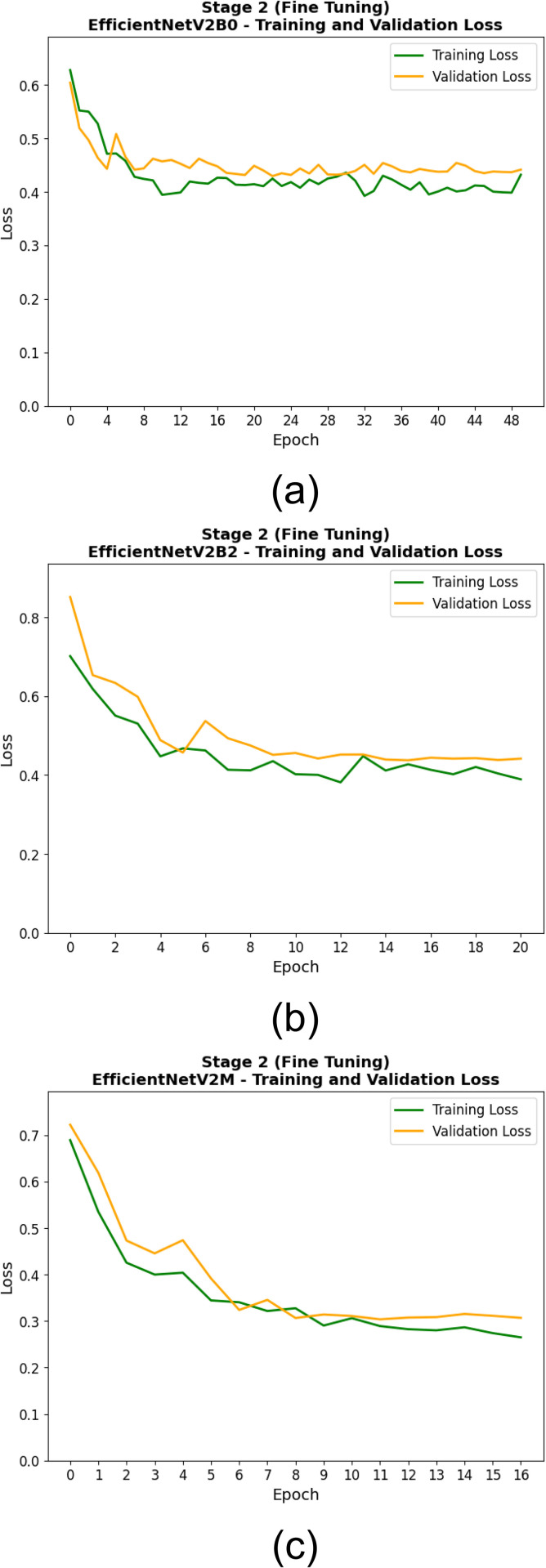
Training and Validation Loss Curves During the Second Stage (Fine-Tuning) for (a) EfficientNetV2B0, (b) EfficientNetV2B2, and (c) EfficientNetV2M Models.

The training processes of the EfficientNetV2 models in the two training stages are shown in Fig 10, Fig 11, Fig 12, and Fig 13. At this first stage (feature extraction), the training and validation accuracy curves are illustrated in Fig 10. Throughout the training, all models progressively converged, with EfficientNetV2M showing the least variance and best overall accuracy. It can further be seen from the loss curves in Fig 11 that EfficientNetV2M has the steepest drop-off and results in the lowest final validation loss, suggesting on the other hand more effective feature learning. In the second stage (fine-tuning), the performance gains are even more substantial. Indeed, and as shown in Fig 12, EfficientNetV2M is still the only model that shows a smooth and consistent upward curve, independent of re-parameterization, suggestive of stable fine-tuning (over fitting comes typically after, giving power-to-fine-tune). Fig 13 uniquely depicts that EfficientNetV2M achieves the lowest training and validation losses, signifying its robustness and superior convergence behavior. These figures together show the learning behavior of EfficientNetV2M and its superior generalization across both stages.

**Fig 14:**
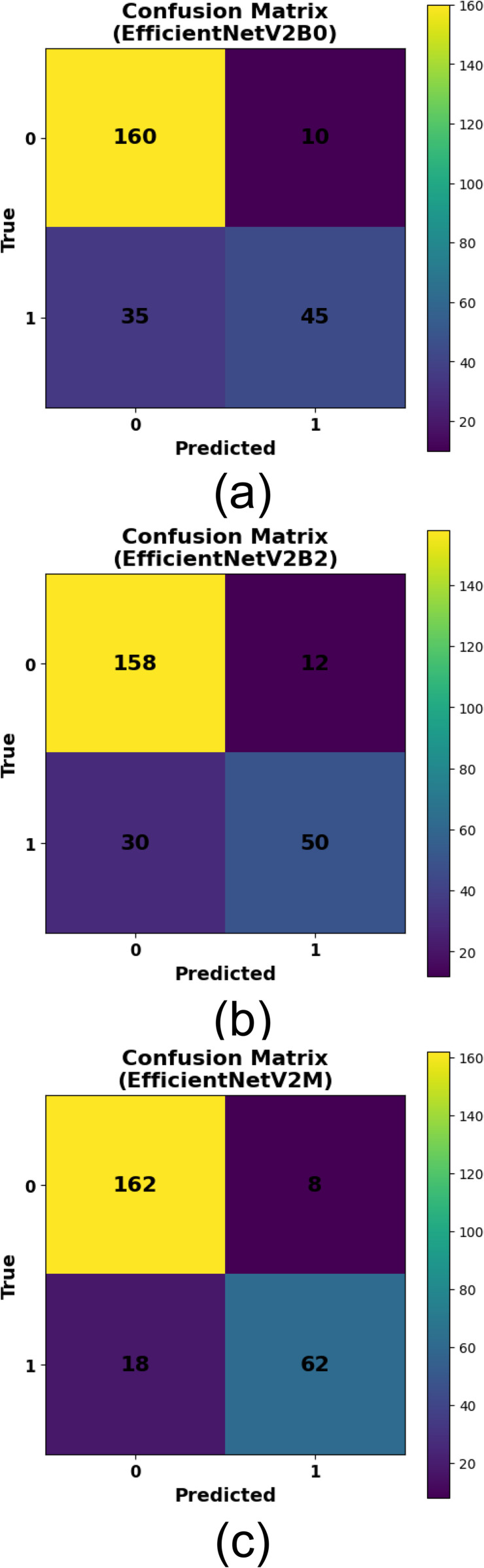
Confusion Matrices for Binary Classification Performance of (a) EfficientNetV2B0, (b) EfficientNetV2B2, and (c) EfficientNetV2M Models on Photovoltaic Cell Fault Detection.

Fig 14 displays the confusion matrices for the EfficientNetV2 models, offering a comprehensive analysis of their classification efficacy in identifying flaws in PV cells. Fig 14(a) demonstrates that EfficientNetV2B0 accurately categorised 160 non-faulty (class 0) and 45 faulty (class 1) samples, while erroneously identifying 10 non-faulty and 35 faulty examples. EfficientNetV2B2, seen in Fig 14(b), enhances performance by accurately recognizing 158 non-defective and 50 faulty samples, resulting in 12 false positives and 30 false negatives. EfficientNetV2M notably attained the optimal results, as seen in Fig 14(c), with 162 true negatives and 62 true positives. It documented just 8 erroneous positives and 18 false negatives, signifying exceptional accuracy and recall. The matrices validate the efficacy of EfficientNetV2M in differentiating between defective and non-defective cells, aligning with its exceptional performance in other assessment criteria.

Although the EfficientNetV2 models perform well overall, Fig 14 shows that the main difficulty is missing defective cells (false negatives), especially for EfficientNetV2B0 (35) and EfficientNetV2B2 (30), with fewer misses for EfficientNetV2M (18). These errors likely occur when faults appear subtle or low-contrast in EL images—such as fine microcracks, thin early-stage snail-trail patterns, or small localized degradation near edges/busbars—which can resemble normal texture or be partially masked by noise and illumination variability.

In order to improve the efficiency and reliability, it is highly essential to investigate renewable energy systems’ challenges. Global sustainability is closely related to the renewable sources; solar and wind ones are designed to provide clean, reliable, and cost-effective alternatives to fossil fuels. These technologies help to avoid greenhouse gas emissions, and reduce the impact on the climate changes. As a result, energy systems’ security and economic development are achieved as well as sustainable energy supply for the future generations is guaranteed [42–48].

While the EfficientNetV2 model family is performing well in general, especially EfficientNetV2M, this research can also look at the sources of the prediction errors. Various factors such as high intra-class variability and low inter-class variations for certain fault classes such as microcracks and snail tails, which may be visually similar to normal texture under different illumination conditions, can lead to these errors. Overall, even though class weighting was used to partially counter the dataset imbalance, this may still have impacted the model ability to learn minority class patterns fully. Discriminability could also be compromised from environmental artifacts, image noise, and overlapping features of monocrystalline and polycrystalline cell defects. Lastly, it is possible in some Grad-CAM visualizations, that the model concentrates on the non-significant regions, suggesting a possible enhancement to localizing or attention methods in future research.

## 4. Conclusion

Overall, this work has effectively shown the utilization of the EfficientNetV2 family, specifically EfficientNetV2B0, EfficientNetV2B2, and EfficientNetV2M, architectures in identifying and categorizing defects in PV cells. The study successfully used DL to reach a high level of accuracy in recognizing several fault types in solar panels, including microcracks, delamination, and soiling. This technique successfully decreased the occurrence of both incorrect positive and incorrect negative results, hence enhancing the dependability of problem detection. Optimizing the operational efficiency and longevity of solar panels is crucial. This study improved the model to address the complex visual features of solar cell images by using techniques like data augmentation to strengthen its capacity to manage diverse environmental conditions. In addition, this study optimized the model’s performance by modifying hyperparameters and refining feature extraction methods. The integration of this model into existing PV monitoring systems enhances the efficiency of predictive maintenance methods. By enabling the prompt detection and categorization of defects, this facilitates the prevention of harm and the extension of the lifespan of installations. Furthermore, the model’s aptitude in handling speed and allocation of resources makes it well-suited for extensive adoption, thereby fulfilling essential needs in the renewable energy sector. As observed from the results, EfficientNetV2M gave the maximum accuracy of 89.6%, but also a maximum precision of 88.6%, recall of 77.5% and F1-score of 82.7%, indicating great fault detection power of the model in the case of PV cells. Second, in order to enhance the generalization, this study utilized a two-stage transfer learning approach, and third, this study utilized the Grad-CAM visualizations to ensure that only the relevant regions corresponding to the defect have been highlighted. The EfficientNetV2B2 exhibited stability in validation performance and would be an appropriate choice where resources are limited, which suits it for applications with limited resources. The model benefitted from these implementations, which dealt with imbalanced data (class weighting) and a context-driven set of auxiliary cell-type inputs. This finding further validates the feasibility of deploying the model in a real-world scenario for the continuous monitoring and predictive maintenance of solar panels. This study not only improves the domains of ML and solar system maintenance, but also offers a framework for future integration of powerful AI technologies into renewable energy management. Such results could indicate a more positive path for sustained elastic energy generation in global solar farms, making them more efficient and ultimately desirable in the long term. An initial step this study suggests for improvement of defect detection systems is to consider transforming the real-time data generally available about the objects of interest into useful information to be studied using ensemble learning techniques. It would reduce both the accuracy and reliability of the systems.

## Supporting information

S1 File Codes for EfficientNetV2B0, EfficientNetV2B2, and EfficientNetV2M models used in the experiment. (RAR)

## Abbreviations

AI: Artificial Intelligence
API: Application Programming Interface
CNN: Convolutional Neural Network
DL: Deep Learning
EL: Electroluminescence
GAN: Generative Adversarial Network
GAP: Global Average Pooling
GradCAM: Gradient-weighted Class Activation Mapping
IoT: Internet of Things
I-V: Current-Voltage
ML: Machine Learning
PV: Photovoltaic
ReLU: Rectified Linear Unit
ResNet: Residual Network
RF: Random Forest
RGB: Red, Green, Blue
SSD: Single Shot MultiBox Detector
VGG: Visual Geometry Group
YOLO: You Only Look Once.
